# Research on the Macroscopic Mechanical Property Continuum of Square Lattices Composed of Piezoelectric Laminated Zigzag Beams

**DOI:** 10.3390/ma18153499

**Published:** 2025-07-25

**Authors:** Zengshuo Zhang, Jinxing Liu

**Affiliations:** Faculty of Civil Engineering and Mechanics, Jiangsu University, Zhenjiang 212013, China; 2212223006@stmail.ujs.edu.cn

**Keywords:** lattice, piezoelectric laminated beam, macroscopic mechanical properties, tailoring of effective moduli

## Abstract

A novel square lattice composed of piezoelectric laminated zigzag beams positioned between each pair of adjacent nodes is proposed. Each zigzag beam is made of four piezoelectric laminated straight beams, formed by laminating a piezoelectric layer and a base layer. The effective moduli are derived by analyzing the unit cell subjected to a stress field. Voltages applied to the piezoelectric layers can be adjusted to tailor the effective moduli of the lattice without altering the microstructure. Theoretical predictions were verified by finite element simulations. Parametric analyses were conducted to examine the effects of voltage on the tailoring of effective moduli in the piezoelectric laminated zigzag beam-based square lattices.

## 1. Introduction

With the continuous development of science and technology, researchers have designed metamaterials [1,2] to meet the increasingly stringent requirements for material properties. Among them, lattice materials usually possess advantages such as high strength [3,4,5], a negative Poisson’s ratio [6,7], negative stiffness [8,9], and adjustability [10,11]. These extraordinary mechanical properties have attracted extensive attention and are applied in modern engineering. In the performance regulation of lattice materials, their unique periodic topological configuration determines that the unit cell serves as the basic unit [12,13] for performance regulation. In the design of the unit cell structure of the lattice, methods such as multi-materials [14,15,16], multi-segment beams [17], and special structureal beams [18,19,20] are widely used. Among them, the macroscopic deformation of the lattice with a chiral structure [21] usually has a coupling effect. For different forms of chiral structures [22,23], further optimizations have been achieved.

Therefore, structural design occupies a core position. However, if the unit cells of the lattice are designed before the lattice is manufactured, when the external load conditions change, they will be unable to adapt to the working environment in an optimal state. We hope that after lattice materials are designed and manufactured, they still have the ability to actively regulate their mechanical properties. Therefore, piezoelectric materials [24,25,26], magnetic materials [27,28,29], and pneumatic structures [30,31,32,33] have been ingeniously applied to achieve the active regulation of the properties of lattice materials.

Among various active mechanical metamaterials, voltage-driven metamaterials are famous for their adjustability and simplicity. By simply changing the applied voltage, their mechanical properties can be effectively regulated [34,35]. The piezoelectric hexagonal lattice [36] composed of piezoelectric beams formed by laminating piezoelectric materials and substrate materials can actively adjust the Young’s modulus, Poisson’s ratio, and shear modulus of the lattice material by applying voltage. Researchers studied the voltage-dependent elastic properties of a new type of piezoelectric composite material [37]. The strain and Young’s modulus can be actively adjusted by regulating the voltage. They also investigated the influence of the inclination angle and thickness of the piezoelectric beam on its elastic properties. In addition, piezoelectric materials have been widely used in many aspects, such as piezoelectric actuators [38], programmable gradient piezoelectric metamaterial beams [39], and so on. These studies have provided useful references for our design.

In the process of deriving the macroscopic modulus of the lattice, the special force field method [40,41,42] and the principle of strain energy equivalence [17,43] are commonly used research approaches. Since the lattice has a periodic structure, we can utilize the structure of a single unit cell to derive the macroscopic modulus of the lattice. Scholars have obtained the stiffness matrix [34] of piezoelectric laminated straight beams using Hamilton’s principle, and they obtained the stiffness matrix of zigzag beams [18] made of homogeneous materials using the equilibrium method [44,45]. In this paper, we combine the two methods and make optimizations, which provide a theoretical basis for this paper.

Our purpose is to propose novel planar lattices with a broad tailoring range of effective moduli composed of piezoelectric components. We aim to provide a new method for the design of voltage-driven metamaterials, thereby advancing their applications in smart structures and tunable materials. The innovation of this paper is that, in contrast to the previous approach of regulating the macroscopic modulus of lattices by changing their microstructure, this paper realizes the regulation of the macroscopic modulus of lattice materials using voltage without altering the lattice’s microstructure. Due to the adjustability of lattices, they will have application value in many fields, such as architectural bridges, structural health monitoring, biomedical devices, electronic sensors [46,47], etc. This paper is structured as follows. In Section 2, a square lattice is designed, which is composed of piezoelectric laminated zigzag beams. A piezoelectric laminated zigzag beam consisting of four piezoelectric laminated straight beams, where the voltage can be adjusted, allows the effective macroscopic moduli of the lattice to be programmable. We derived the relationship between the nodal displacements of the piezoelectric laminated zigzag beam and the voltage or load. In Section 3, effective macroscopic moduli of square lattices composed of piezoelectric laminated zigzag beams are obtained through the special force field method. In Section 4, the results obtained by the method we adopted are compared with the results of finite element simulations, showing good agreement when the voltage changes, and the existing differences are also analyzed. In Section 5, we analyze the influence of voltage and external stress on the macroscopic moduli of the lattice, including Young’s modulus, Poisson’s ratio, and the shear modulus. We also explain the regulation mechanism by which voltage regulates their behavior. This paper ends with conclusions in Section 6.

## 2. Square Lattices Composed of Piezoelectric Laminated Zigzag Beams

**Figure 1:**
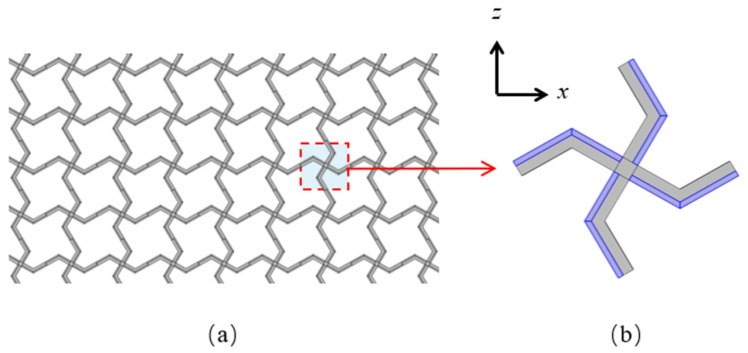
The square lattice composed of the piezoelectric laminated zigzag beams. (**a**) Structure of lattice; (**b**) its unit cell.

**Figure 2:**
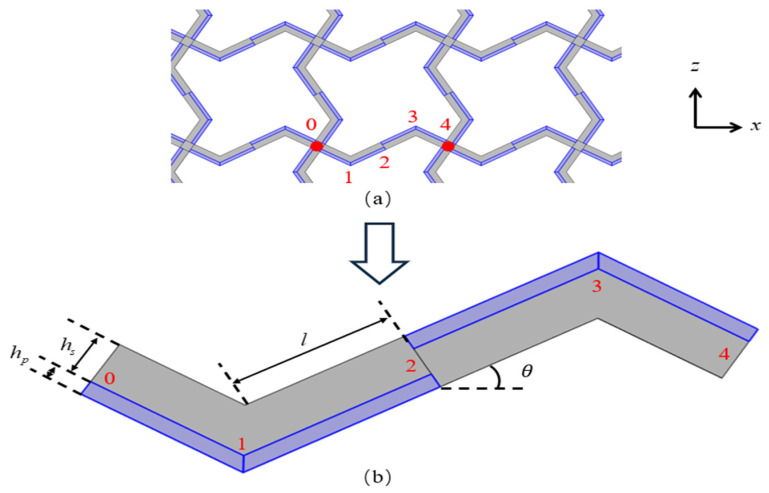
The square lattice composed of the piezoelectric laminated zigzag beams: (**a**) structure; (**b**) dimensions.

A schematic diagram of the square lattices and their unit cell are shown in Figure 1. The center points of each pair of adjacent unit cells are connected by a piezoelectric laminated zigzag beam. For example, the nodes from 0 to 4 in Figure 2a are connected by such a beam, which is composed of four piezoelectric laminated straight beams arranged at specific angles. The dimensions of each straight beam are marked in Figure 2b. Since the lattice has a periodic structure, we can represent the deformation of the lattice by the deformation of these piezoelectric laminated zigzag beams, so we need to obtain the stiffness matrix of the two nodes of this piezoelectric laminated zigzag beam, which can be obtained based on the stiffness matrix of the piezoelectric laminated straight beam that composes it.

**Figure 3:**
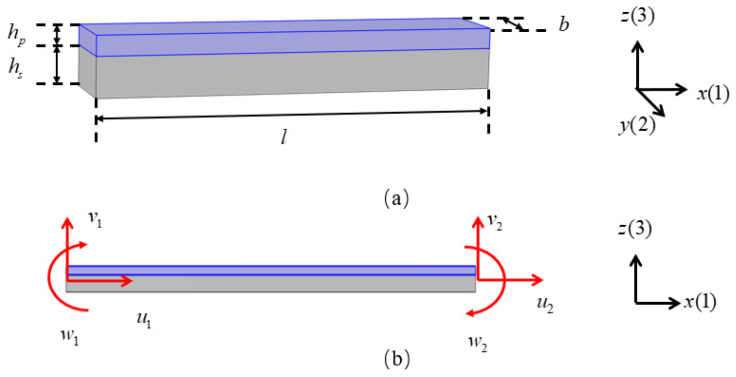
Piezoelectric laminated straight beam: (**a**) the dimensions of the piezoelectric laminated beam; (**b**) double-node piezoelectric laminated beam.

**Figure 4:**
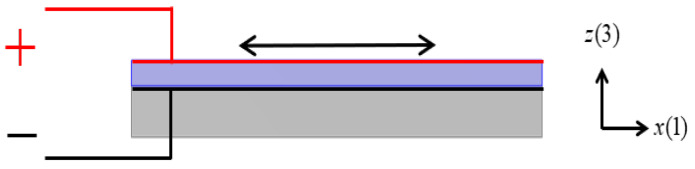
The method of applying voltage to piezoelectric laminated beam.

The schematic diagram of the piezoelectric laminated straight beam and its dimensions are shown in Figure 3a, which consists of a piezoelectric layer (blue) on the top and a layer (grey) on the bottom. The piezoelectric material is PZT-5H, and the substrate material is aluminum. A cross-section in the *xz* plane is shown in Figure 3b. When *b* is sufficiently large, the influence of the width is negligible, allowing the structural analysis to be simplified to a plane strain condition. The voltage is applied to each segment of the piezoelectric laminated beam in the manner shown in Figure 4 for overall voltage regulation. The red line represents the positive terminal of the voltage, and the black line represents the negative terminal of the voltage. When voltage is applied in this way, the piezoelectric layer will undergo tensile deformation. When the piezoelectric laminated straight beams are assembled into a lattice and rotated, the interface between the two materials remains grounded, and the surface of the piezoelectric layer away from the base layer serves as the positive pole of the power supply.

Next, we will derive the stiffness matrix of the piezoelectric laminated straight beam [34]. To simplify the calculation, the beam with the $d_{31}$ (i.e., when the electric field is applied in direction 3, induced strain develops in direction 1) piezoelectric patch attached on its surface is considered. In this paper, directions 1, 2, and 3 of the coordinate system are the same as those of the *x*, *y*, and *z* axes. Suppose that the axial displacement and lateral displacement of the beam [48] are

(1) $$\begin{array}{c} u\left(x,z,t\right)=u^{0}\left(x,y\right)-{zw}_{,x}^{0}\left(x,t\right)\\ w\left(x,t\right)=w^{0}\left(x,y\right) \end{array},$$

where $u^{0}\left(x,t\right)$ and $w^{0}\left(x,t\right)$ represent the displacements of the neutral axis of the beam at an arbitrary point *x* in the axial and lateral directions at time *t*. The total potential energy of the piezoelectric laminated beam is the sum of the elastic potential energy of the substrate beam and the elastic potential energy of the piezoelectric layer, which can be written as

(2) $$\psi =\frac{1}{2}\left(\int_{V_{s}}{\left\{\varepsilon \right\}}^{t}\left\{\sigma^{s}\right\}dV_{s}+\int_{V_{p}}{\left\{\varepsilon \right\}}^{t}\left\{\sigma^{p}\right\}dV_{p}\right),$$

In the above formula, ε is the strain and σ is the stress, The “*s*” and “*p*” in the subscript or superscript represent the substrate and the piezoelectric material, respectively. The volume of each material is obtained by integrating individual volume elements. The superscript “*t*” represents the matrix transpose. Then, the strain in direction 1 is

(3) $$S_{11}\left(x,z,t\right)=\frac{\partial u^{0}\left(x,t\right)}{\partial x}-z\frac{\partial^{2}w^{0}\left(x,t\right)}{\partial x^{2}}.$$

Assume that the material of the substrate layer is isotropic and obeys Hooke’s law; then, the stress in direction 1 of the substrate layer is

(4) $$\sigma_{11}^{s}\left(x,z,t\right)=E_{s}\varepsilon_{11}\left(x,z,t\right),$$

where $E_{s}$ represents the elastic modulus of the substrate layer material; then, the elastic potential energy of the substrate layer is

(5) $$\begin{array}{cc} \int_{V_{s}}{\left\{\varepsilon \right\}}^{t}\left\{\sigma^{s}\right\}dV_{s} & =\frac{1}{2}\int_{V_{s}}E_{s}\varepsilon_{11}\left(x,z,t\right)dV_{s}\\ & =\frac{1}{2}\int_{0}^{l}E_{s}\left[\iint_{s}\left[\begin{array}{l} {\left(\frac{\partial u^{0}\left(x,t\right)}{\partial x}\right)}^{2}+z^{2}{\left(\frac{\partial^{2}w^{0}\left(x,t\right)}{\partial x^{2}}\right)}^{2}\\ -2z\frac{\partial u^{0}\left(x,t\right)}{\partial x}\frac{\partial^{2}w^{0}\left(x,t\right)}{\partial x^{2}} \end{array}\right]ds\right]dx. \end{array}$$

The stress in direction 1 of the piezoelectric layer is expressed as

(6) $$\sigma_{11}^{p}\left(x,z,t\right)=E_{p}\varepsilon_{11}-{\bar{e}}_{31}E_{3}=E_{p}\varepsilon_{11}\left(x,z,t\right)+{\bar{e}}_{31}\frac{U}{h_{p}},$$

where the electric field strength is $E_{3}=-U/h_{p}$ (i.e., the electric field can be written as a function of the voltage). Among them, the applied voltage is represented by *U*, and the thickness of the piezoelectric material is $h_{p}$. The dielectric constant, elastic modulus, and piezoelectric stress constant of the piezoelectric layer are

(7) $${\bar{\zeta }}_{33}^{\varepsilon }=\zeta_{33}^{\sigma }-\frac{d_{31}^{2}}{s_{11}^{E}},E_{p}=\frac{1}{s_{11}^{E}},{\bar{e}}_{31}=\frac{d_{31}}{s_{11}^{E}},$$

The elastic potential energy of the piezoelectric layer is

(8) $$\begin{array}{cc} \int_{V_{p}}{\left\{\varepsilon \right\}}^{t}\left\{\sigma^{p}\right\}dV_{p} & =\frac{1}{2}\int_{V_{s}}E_{s}{\varepsilon_{11}}^{2}\left(x,z,t\right)+{\bar{e}}_{31}\frac{U}{h_{p}}S_{11}\left(x,z,t\right)dV_{p}\\ & =\frac{1}{2}\int_{0}^{l}E_{s}\left\{\iint_{p}\left[\begin{array}{l} {\left(\frac{\partial u^{0}\left(x,t\right)}{\partial x}\right)}^{2}+z^{2}{\left(\frac{\partial^{2}w^{0}\left(x,t\right)}{\partial x^{2}}\right)}^{2}\\ -2z\frac{\partial u^{0}\left(x,t\right)}{\partial x}\frac{\partial^{2}w^{0}\left(x,t\right)}{\partial x^{2}} \end{array}\right]ds\right\}\\ & +\left[U\iint_{p}\frac{{\bar{e}}_{31}}{h_{p}}\frac{\partial u^{0}\left(x,t\right)}{\partial x}ds-U\iint_{p}z\frac{{\bar{e}}_{31}}{h_{p}}\frac{\partial^{2}w^{0}\left(x,t\right)}{\partial x^{2}}ds\right]dx, \end{array}$$

Let $\left(A_{s},H_{s,},I_{s}\right)=\iint_{s}\left(1,z,z^{2}\right)dydz$, $\left(A_{p},H_{p,},I_{p}\right)=\iint_{p}\left(1,z,z^{2}\right)dydz$, $B_{p}=\iint_{p}\frac{{\bar{e}}_{31}}{h_{p}}dydz$, and $J_{p}=\iint_{p}z\frac{{\bar{e}}_{31}}{h_{p}}dydz$; then, the elastic potential energy of the piezoelectric laminated straight beam can be written as

(9) $$\psi =\frac{1}{2}\int_{0}^{l}\left\{\begin{array}{l} E_{s}\left[A_{s}{\left(\frac{\partial u^{0}\left(x,t\right)}{\partial x}\right)}^{2}+I_{s}{\left(\frac{\partial^{2}w^{0}\left(x,t\right)}{\partial x^{2}}\right)}^{2}-2H_{s}\frac{\partial u^{0}\left(x,t\right)}{\partial x}\frac{\partial^{2}w^{0}\left(x,t\right)}{\partial x^{2}}\right]\\ +E_{p}\left[A_{p}{\left(\frac{\partial u^{0}\left(x,t\right)}{\partial x}\right)}^{2}+I_{p}{\left(\frac{\partial^{2}w^{0}\left(x,t\right)}{\partial x^{2}}\right)}^{2}-2H_{p}\frac{\partial u^{0}\left(x,t\right)}{\partial x}\frac{\partial^{2}w^{0}\left(x,t\right)}{\partial x^{2}}\right]\\ +B_{p}U\frac{\partial u^{0}\left(x,t\right)}{\partial x}-J_{p}U\frac{\partial^{2}w^{0}\left(x,t\right)}{\partial x^{2}} \end{array}\right\}dx.$$

The internal electrical energy [48] in the piezoelectric layer can be written as

(10) $$\begin{array}{cc} W_{ie} & =\frac{1}{2}{\int_{V_{p}}\left\{E\right\}}^{t}\left\{D\right\}dV_{p}\\ & =-\frac{1}{2}\int_{0}^{l}\left[B_{p}U\frac{\partial u^{0}\left(x,t\right)}{\partial x}-J_{p}U\frac{\partial^{2}w^{0}\left(x,t\right)}{\partial x^{2}}\right]dx+\frac{1}{2}C_{p}U^{2}, \end{array}$$

where $C_{p}={\bar{\zeta }}_{33}^{\varepsilon }\frac{A_{p}}{h_{p}}$, D represents the electric displacement.

We consider a two-node Euler beam element with three degrees of freedom (i.e., axial, lateral, rotational) at each node. Its unit length is 1, and the displacement vector q can be expressed as $\left[q\right]={\left[\begin{array}{cccccc} u_{1} & v_{1} & w_{1} & u_{2} & v_{2} & w_{2} \end{array}\right]}^{t}$. The values of the displacements $u^{0}$ and $v^{0}$ can be expressed using the relations given bellowing:

(11) $$\left[\begin{array}{l} u^{0}\\ v^{0} \end{array}\right]=\left[\begin{array}{cccccc} F_{1} & 0 & 0 & F_{2} & 0 & 0\\ 0 & H_{1} & H_{2} & 0 & H_{3} & H_{4} \end{array}\right]\left[q\right],$$

where the shape function matrix is

(12) $$\left[A_{q}\right]=\left[\begin{array}{cccccc} F_{1} & 0 & 0 & F_{2} & 0 & 0\\ 0 & H_{1} & H_{2} & 0 & H_{3} & H_{4} \end{array}\right],$$

Then, we have

(13) $$\begin{array}{l} F_{1}=1-\xi ,\\ F_{2}=\xi ,\\ H_{1}=1-3\xi^{2}+2\xi^{3},\\ H_{2}=l\left(\xi -2\xi^{2}+\xi^{3}\right),\\ H_{3}=3\xi^{2}-2\xi^{3},\\ H_{4}=l\left(-\xi^{2}+\xi^{3}\right), \end{array}$$

where ξ=x/l; then, Equation (11) can be abbreviated as

(14) $$\begin{array}{c} u^{0}=\left[n_{1}\right]\left[A_{q}\right]\left[q\right],\\ w^{0}=\left[n_{2}\right]\left[A_{q}\right]\left[q\right], \end{array}$$

where $\left[n_{1}\right]=\left[1,0\right]$, $\left[n_{2}\right]=\left[0,1\right]$. Taking the variational and differentiating Equation (14), we get

(15) $$\begin{array}{c} \delta u_{,x}^{0}={\left[B_{1}\right]}_{,x}\left[\delta q\right],\\ \delta w_{,xx}^{0}={\left[B_{2}\right]}_{,xx}\left[\delta q\right], \end{array}$$

where the values of $\left[B_{1}\right]$ and $\left[B_{2}\right]$ are

(16) $$\begin{array}{c} \left[B_{1}\right]=\left[n_{1}\right]\left[A_{q}\right],\\ \left[B_{2}\right]=\left[n_{2}\right]\left[A_{q}\right]. \end{array}$$

Keep the voltage constant and take the variations in potential energy and internal energy. Since we want to deform the structure and keep it in a specific position—that is, to prevent it from vibrating—it degenerates into the principle of minimum potential energy. We obtain the following relationship:

(17) $$\delta \psi ={\left[\delta q\right]}^{t}\left[\begin{array}{l} \left(E_{s}A_{s}+E_{p}A_{p}\right)\left[M_{11}\right]+\left(E_{s}I_{s}+E_{p}I_{p}\right)\left[M_{22}\right]\\ -\left(E_{s}H_{s}+E_{p}H_{p}\right)\left[M_{12}\right]-\left(E_{s}H_{s}+E_{p}H_{p}\right)\left[M_{21}\right] \end{array}\right]\left[q\right]+\left[\eta \right],$$

where

(18) $$\begin{array}{l} \left[M_{11}\right]=\int_{0}^{l}{\left[B_{1}\right]}_{,x}^{t}{\left[B_{1}\right]}_{,x}dx,\left[M_{12}\right]=\int_{0}^{l}{\left[B_{1}\right]}_{,x}^{t}{\left[B_{2}\right]}_{,xx}dx,\\ \left[M_{21}\right]=\int_{0}^{l}{\left[B_{2}\right]}_{,xx}^{t}{\left[B_{1}\right]}_{,x}dx,\left[M_{22}\right]=\int_{0}^{l}{\left[B_{2}\right]}_{,xx}^{t}{\left[B_{2}\right]}_{,xx}dx,\\ \left[\eta_{1}\right]=\int_{0}^{l}\frac{1}{2}B_{p}U{\left[B_{1}\right]}_{,x}^{t}dx,\left[\eta_{2}\right]=\int_{0}^{l}\frac{1}{2}J_{p}U{\left[B_{2}\right]}_{,xx}^{t}dx,\\ \left[\eta \right]=\left[\eta_{1}\right]-\left[\eta_{2}\right]. \end{array}$$

Abbreviate Equation (17) into the following form:

(19) $$\delta \psi ={\left[\delta q\right]}^{t}\left[K_{0}\right]\left[q\right]+\left[\eta \right],$$

where the value of $\left[K_{0}\right]$ is

(20) $$\left[K_{0}\right]=\left[\begin{array}{l} \left(E_{s}A_{s}+E_{p}A_{p}\right)\left[M_{11}\right]+\left(E_{s}I_{s}+E_{p}I_{p}\right)\left[M_{22}\right]\\ -\left(E_{s}H_{s}+E_{p}H_{p}\right)\left[M_{12}\right]-\left(E_{s}H_{s}+E_{p}H_{p}\right)\left[M_{21}\right] \end{array}\right].$$

Take the variation in the electric potential energy; we get

(21) $$\delta W_{ie}=-{\left[\delta q\right]}^{t}\left[\eta \right],$$

By using Hamilton’s principle, we have

(22) $$\int_{t_{1}}^{t_{2}}\left(\delta \psi -\delta W_{ie}\right)dt=0,$$

Substitute Equations (19) and (21) into Equation (22), we get

(23) $$\int_{t_{1}}^{t_{2}}{\left[\delta q\right]}^{t}\left(\left[K_{0}\right]\left[q\right]+2\left[\eta \right]\right)dt=\left[0\right],$$

The final form of the equilibrium equation is

(24) $$\left[K_{0}\right]\left[q\right]+2\left[\eta \right]=\left[0\right],$$

Let $\left[F\right]=-2\left[\eta \right]$; the relationship between the nodal displacements and the voltage of a piezoelectric laminated straight beam can be written as

(25) $$\left[K_{0}\right]\left[q\right]=\left[F\right],$$

Then, the displacement of the piezoelectric laminated beam caused by the change in voltage can be calculated by the following equation:

(26) $${\left[q\right]}_{6\times 1}={\left[K_{0}\right]}_{6\times 6}^{-1}{\left[F\right]}_{6\times 1},$$

where the expressions of the stiffness matrix $\left[K_{0}\right]$ and the force vector $\left[F\right]$ are

(27) $$\left[K_{0}\right]=\left[\begin{array}{cccccc} A & 0 & -B & -A & 0 & B\\ 0 & 12C & 6Cl & 0 & -12C & 6Cl\\ -B & 6Cl & 4Cl^{2} & B & -6Cl & 2Cl^{2}\\ -A & 0 & B & A & 0 & -B\\ 0 & -12C & -6Cl & 0 & 12C & -6Cl\\ B & 6Cl & 2Cl^{2} & -B & -6Cl & 4Cl^{2} \end{array}\right],$$

(28) $$\left[F\right]={\left[\begin{array}{cccccc} B_{p}U & 0 & -J_{p}U & -B_{p}U & 0 & J_{p}U \end{array}\right]}^{t},$$

The values of A, B, and C are

(29) $$A=\frac{E_{s}A_{s}+E_{p}A_{p}}{l},B=\frac{E_{s}H_{s}+E_{p}H_{p}}{l},C=\frac{E_{s}I_{s}+E_{p}I_{p}}{l^{3}}.$$

**Figure 5:**
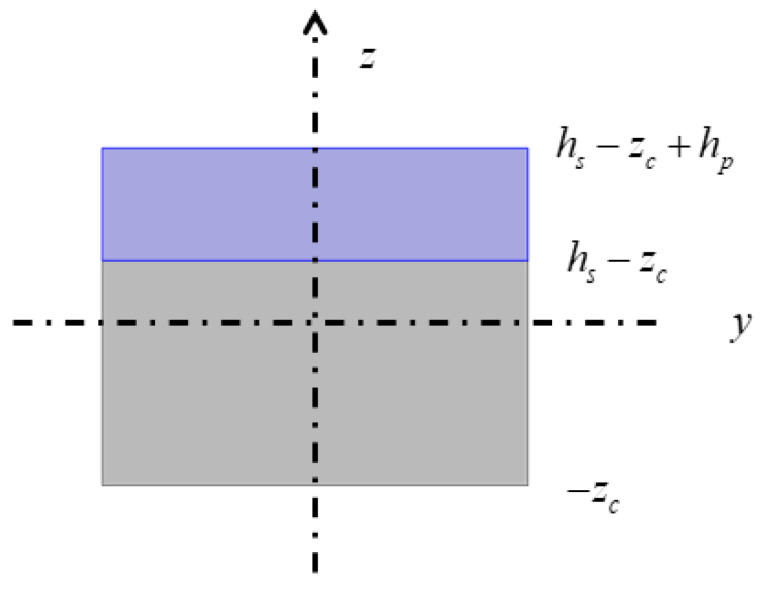
The *yz* cross-section of the piezoelectric laminated beam.

Now, determine the values of coefficients of each term in Equation (26). Figure 5 shows the cross-section of a piezoelectric laminated straight beam, and the position of its neutral axis is given by the following formula.

(30) $$z_{c}=\frac{E_{p}h_{p}\left(\frac{h_{p}}{2}+h_{s}\right)+E_{s}h_{s}\frac{h_{s}}{2}}{E_{s}h_{s}+E_{p}h_{p}}.$$

The area of the substrate layer is given as

(31) $$A_{s}=\int_{0}^{b}\int_{-z_{c}}^{h_{s}-z_{c}}dydz=bh_{s},$$

The first moment of the substrate layer is given as

(32) $$H_{s}=\int_{0}^{b}\int_{-z_{c}}^{h_{s}-z_{c}}zdydz=\frac{1}{2}bh_{s}(h_{s}-2z_{c}),$$

The second moment of the substrate layer is given as

(33) $$I_{s}=\int_{0}^{b}\int_{-z_{c}}^{h_{s}-z_{c}}z^{2}dydz=\frac{1}{3}bh_{s}\left(h_{s}^{2}-3h_{s}z_{c}+3z_{c}^{2}\right),$$

The area of the piezoelectric layer is given as

(34) $$A_{p}=\int_{0}^{b}\int_{h_{s}-z_{c}}^{h_{s}-z_{c}+h_{p}}dydz=bh_{p},$$

The first moment of the piezoelectric layer is given as

(35) $$H_{p}=\int_{0}^{b}\int_{h_{s}-z_{c}}^{h_{s}-z_{c}+h_{p}}zdydz=\frac{1}{2}bh_{p}\left[h_{p}+2\left(h_{s}-z_{c}\right)\right],$$

The second moment of the piezoelectric layer is given as

(36) $$I_{p}=\int_{0}^{b}\int_{h_{s}-z_{c}}^{h_{s}-z_{c}+h_{p}}z^{2}dydz=\frac{1}{3}bh_{p}\left[3{\left(h_{s}-z_{c}\right)}^{2}+3\left(h_{s}-z_{c}\right)h_{p}+h_{p}^{2}\right],$$

Piezoelectric coupling terms are given as

(37) $$B_{p}=\int_{0}^{b}\int_{h_{s}-z_{c}}^{h_{s}-z_{c}+h_{p}}\frac{e_{31}}{h_{p}}dydz=d_{31}E_{p}b,$$

(38) $$J_{p}=\int_{0}^{b}\int_{h_{s}-z_{c}}^{h_{s}-z_{c}+h_{p}}\frac{e_{31}}{h_{p}}zdydz=\frac{d_{31}E_{p}b}{2}\left[h_{p}+2\left(h_{s}-z_{c}\right)\right].$$

Now, we use four segments of the above mentioned piezoelectric laminated straight beams to form a piezoelectric laminated zigzag beam. The stiffness matrix of the piezoelectric laminated zigzag beam can be obtained based on the stiffness matrix of the piezoelectric laminated straight beam composing it. For a piezoelectric laminated straight beam, the relationship between voltage and displacement in the local coordinate system can be expressed as Equation (25). Then, the stiffness matrix of the piezoelectric laminated straight beam in the global coordinate system can be expressed as

(39) $$\left[K_{ij}\right]=\left[T_{ij}\right]\left[K_{0}\right]{\left[T_{ij}\right]}^{t},$$

where $\left[K_{0}\right]$ represents the stiffness matrix of the piezoelectric laminated straight beam in the local coordinate system, and i and j are the numbering of the two ending joints, respectively. $\left[T_{ij}\right]$ is the transformation matrix from the local to the global coordinate system, and it can be expressed as

(40) $$\left[T_{ij}\right]=\left[\begin{array}{cccccc} \cos\beta_{ij} & \sin\beta_{ij} & 0 & 0 & 0 & 0\\ -\sin\beta_{ij} & \cos\beta_{ij} & 0 & 0 & 0 & 0\\ 0 & 0 & 1 & 0 & 0 & 0\\ 0 & 0 & 0 & \cos\beta_{ij} & \sin\beta_{ij} & 0\\ 0 & 0 & 0 & -\sin\beta_{ij} & \cos\beta_{ij} & 0\\ 0 & 0 & 0 & 0 & 0 & 1 \end{array}\right],$$

where $\beta_{ij}$ is the transformation angle of the piezoelectric laminated straight beam between nodes i and j from the local coordinate system to the global coordinate system.

The relationship between the node displacements and the voltage of the piezoelectric laminated straight beam of nodes i and j in the global coordinate system can be expressed as

(41) $$\left[K_{ij}\right]\left[d_{ij}\right]=\left[F_{ij}^{U}\right],$$

where $\left[d_{ij}\right]$ and $\left[F_{ij}^{U}\right]$ represent the displacement vectors and force caused by voltage in the global coordinate system, and $\left[F_{ij}^{U}\right]$ can be expressed as

(42) $$\left[F_{ij}^{U}\right]={\left[T_{ij}\right]}^{t}\left[F_{ij}\right],$$

where $\left[F_{ij}\right]$ is denoted in Equation (28).

**Figure 6:**
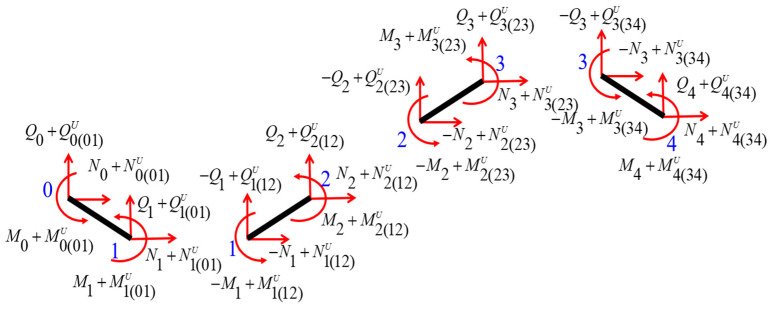
Force analysis of a four-segment piezoelectric laminated zigzag beam.

For the piezoelectric laminated zigzag beam in Figure 6, the stiffness matrices of four piezoelectric laminated straight beams can be expressed as

(43) $$\left[\begin{array}{cc} K_{01}^{1} & K_{01}^{2}\\ K_{01}^{3} & K_{01}^{4} \end{array}\right]\left[\begin{array}{c} d_{0}\\ d_{1} \end{array}\right]=\left[\begin{array}{c} F_{0}\\ F_{1} \end{array}\right]+\left[\begin{array}{c} F_{0(01)}^{U}\\ F_{1(01)}^{U} \end{array}\right],$$

(44) $$\left[\begin{array}{cc} K_{12}^{1} & K_{12}^{2}\\ K_{12}^{3} & K_{12}^{4} \end{array}\right]\left[\begin{array}{c} d_{1}\\ d_{2} \end{array}\right]=\left[\begin{array}{c} -F_{1}\\ F_{2} \end{array}\right]+\left[\begin{array}{c} F_{1(12)}^{U}\\ F_{2(12)}^{U} \end{array}\right],$$

(45) $$\left[\begin{array}{cc} K_{23}^{1} & K_{23}^{2}\\ K_{23}^{3} & K_{23}^{4} \end{array}\right]\left[\begin{array}{c} d_{2}\\ d_{3} \end{array}\right]=\left[\begin{array}{c} -F_{2}\\ F_{3} \end{array}\right]+\left[\begin{array}{c} F_{2(23)}^{U}\\ F_{3(23)}^{U} \end{array}\right],$$

(46) $$\left[\begin{array}{cc} K_{34}^{1} & K_{34}^{2}\\ K_{34}^{3} & K_{34}^{4} \end{array}\right]\left[\begin{array}{c} d_{3}\\ d_{4} \end{array}\right]=\left[\begin{array}{c} -F_{3}\\ F_{4} \end{array}\right]+\left[\begin{array}{c} F_{3(34)}^{U}\\ F_{4(34)}^{U} \end{array}\right],$$

where $K_{ij}^{1}$, $K_{ij}^{2}$, $K_{ij}^{3}$, and $K_{ij}^{4}$ are, respectively, the upper-left, upper-right, bottom-left, and bottom-right 3×3 sub-matrices of $K_{ij}$. $F_{0}$ and $F_{1}$ are generalized forces on ends 0 and 1 of piezoelectric laminated straight beam 01; $F_{2}$ and $F_{3}$ are generalized forces, respectively, at end 2 in piezoelectric laminated straight beam 12 and at end 3 in piezoelectric laminated straight beam 23; $F_{4}$ denotes the generalized forces at end 4 in piezoelectric laminated straight beam 34. $F_{i}$(i = 0, 1, 2, 3, 4) = ${\left[\begin{array}{ccc} N_{i} & Q_{i} & M_{i} \end{array}\right]}^{t},$ where $N_{i}$, $Q_{i}$, and $M_{i}$ are the axial, shear forces, and bending moments on joint i. $d_{i}$(i = 0, 1, 2, 3, 4) is the generalized displacement at joint i. $F_{0(01)}^{U}$ and $F_{1(01)}^{U}$ are forces caused by voltage on ends 0 and 1 of piezoelectric laminated straight beam 01; $F_{1(12)}^{U}$ and $F_{2(12)}^{U}$ are forces caused by the voltage on ends 1 and 2 of piezoelectric laminated straight beam 12; $F_{2(23)}^{U}$ and $F_{3(23)}^{U}$ are forces caused by the voltage on ends 2 and 3 of piezoelectric laminated straight beam 23; $F_{3(34)}^{U}$ and $F_{4(34)}^{U}$ are forces caused by the voltage on ends 3 and 4 of piezoelectric laminated straight beam 34; $F_{i(ij)}^{U}$ and $F_{j(ij)}^{U}$ are forces caused by the voltage on ends i and j of piezoelectric laminated straight beam ij. $F_{i(ij)}^{U}\left(i\;=\;0,\;1,\;2,\;3,\;4\right)={\left[\begin{array}{ccc} N_{i(ij)}^{U} & Q_{i(ij)}^{U} & M_{i(ij)}^{U} \end{array}\right]}^{t}$, where $N_{i(ij)}^{U}$, $Q_{i(ij)}^{U}$, and $M_{i(ij)}^{U}$ are the axial, shear forces, and bending moments caused by the voltage on joint i.

According to the balance of the force at node 1, node 2, and node 3, we can get eight equilibrium equations with eight unknowns.

(47) $$F_{0}=K_{01}^{1}d_{0}+K_{01}^{2}d_{1}-F_{0(01)}^{U},$$

(48) $$\left\{\begin{array}{c} F_{1}=K_{01}^{3}d_{0}+K_{01}^{4}d_{1}-F_{1(01)}^{U}=-K_{12}^{1}d_{1}-K_{12}^{2}d_{2}+F_{1(12)}^{U}\\ F_{2}=K_{12}^{3}d_{1}+K_{12}^{4}d_{2}-F_{2(12)}^{U}=-K_{23}^{1}d_{2}-K_{23}^{2}d_{3}+F_{2(23)}^{U}\\ F_{3}=K_{23}^{3}d_{2}+K_{23}^{4}d_{3}-F_{3(23)}^{U}=-K_{34}^{1}d_{3}-K_{34}^{2}d_{4}+F_{3(34)}^{U} \end{array}\right.,$$

(49) $$F_{4}=K_{34}^{3}d_{3}+K_{34}^{4}d_{4}-F_{4(34)}^{U},$$

Solving Equation (48), $d_{1}$, $d_{2}$, and $d_{3}$ can be represented by $d_{0}$ and $d_{4}$, i.e.,

(50) $$\left[\begin{array}{c} d_{1}\\ d_{2}\\ d_{3} \end{array}\right]=P\left[\begin{array}{ccc} -K_{01}^{3} & 0 & 0\\ 0 & 0 & 0\\ 0 & 0 & -K_{34}^{2} \end{array}\right]\left[\begin{array}{c} d_{0}\\ 0\\ d_{4} \end{array}\right]+\left[\begin{array}{c} B_{1}\\ B_{2}\\ B_{3} \end{array}\right],$$

where 0 is a 3×3 zero matrix.

(51) $$P={\left[\begin{array}{ccc} K_{01}^{4}+K_{12}^{1} & K_{12}^{2} & 0\\ K_{12}^{3} & K_{12}^{4}+K_{23}^{1} & K_{23}^{2}\\ 0 & K_{23}^{3} & K_{23}^{4}+K_{34}^{1} \end{array}\right]}^{-1},$$

(52) $$\left[\begin{array}{c} B_{1}\\ B_{2}\\ B_{3} \end{array}\right]=P\left[\begin{array}{c} F_{1(01)}^{U}+F_{1(12)}^{U}\\ F_{2(12)}^{U}+F_{2(23)}^{U}\\ F_{3(23)}^{U}+F_{3(34)}^{U} \end{array}\right].$$

Let the coefficient matrices of $d_{0}$ and $d_{4}$ be represented by A. Then, $d_{1}$, $d_{2}$, and $d_{3}$ can be expressed in terms of $d_{0}$ and $d_{4}$, and they can be written as

(53) $$\left[\begin{array}{c} d_{1}\\ d_{2}\\ d_{3} \end{array}\right]=\left[\begin{array}{cc} A_{11} & A_{12}\\ A_{21} & A_{22}\\ A_{31} & A_{32} \end{array}\right]\left[\begin{array}{c} d_{0}\\ d_{4} \end{array}\right]+\left[\begin{array}{c} B_{1}\\ B_{2}\\ B_{3} \end{array}\right].$$

Substituting Equation (53) into Equations (47) and (49), we get

(54) $$\left[\begin{array}{cc} K_{1} & K_{2}\\ K_{3} & K_{4} \end{array}\right]\left[\begin{array}{c} d_{0}\\ d_{4} \end{array}\right]=\left[\begin{array}{c} F_{0}\\ F_{4} \end{array}\right]+\left[\begin{array}{c} C_{0}\\ C_{4} \end{array}\right],$$

where $C_{0}$ and $C_{4}$ are forces caused by the voltage on nodes 0 and 4 of piezoelectric laminated zigzag beam 04:

(55) $$\begin{array}{c} C_{0}=F_{0(01)}^{U}-K_{01}^{2}B_{1}\\ C_{4}=F_{4(34)}^{U}-K_{34}^{3}B_{3} \end{array},$$

Thus, we have obtained the relationship between the node displacements at both ends of the piezoelectric laminated zigzag beam under the action of the voltage, and it can be expressed as

(56) $$\left[\begin{array}{cc} K_{1} & K_{2}\\ K_{3} & K_{4} \end{array}\right]\left[\begin{array}{c} d_{0}\\ d_{4} \end{array}\right]=\left[\begin{array}{c} C_{0}\\ C_{4} \end{array}\right],$$

where

(57) $$\begin{array}{c} K_{1}=K_{01}^{1}+K_{01}^{2}A_{11}\\ K_{2}=K_{01}^{2}A_{13}\\ K_{3}=K_{34}^{3}A_{31}\\ K_{4}=K_{34}^{4}+K_{34}^{3}A_{33} \end{array}.$$

We denote the above stiffness matrix as $K_{zz}^{U}$, which is symmetric, i.e.,

(58) $$K_{zz}^{U}=\left[\begin{array}{cccccc} k_{11} & k_{12} & k_{13} & -k_{11} & -k_{12} & k_{13}\\ k_{12} & k_{22} & k_{23} & -k_{12} & -k_{22} & k_{23}\\ k_{13} & k_{23} & k_{33} & -k_{13} & -k_{23} & k_{36}\\ -k_{11} & -k_{12} & -k_{13} & k_{11} & k_{12} & -k_{13}\\ -k_{12} & -k_{22} & -k_{23} & k_{12} & k_{22} & -k_{23}\\ k_{13} & k_{23} & k_{36} & -k_{13} & -k_{23} & k_{33} \end{array}\right].$$

When there is no voltage applied, the piezoelectric laminated zigzag beam can be regarded as a composite material beam. By using the same method as in [18], we can obtain the relationship between the node displacements at both ends and the applied forces, which can be expressed as

(59) $$\left[\begin{array}{cc} K_{1}^{f} & K_{1}^{f}\\ K_{1}^{f} & K_{1}^{f} \end{array}\right]\left[\begin{array}{c} d_{0}\\ d_{4} \end{array}\right]=\left[\begin{array}{c} F_{0}\\ F_{4} \end{array}\right],$$

We write the stiffness matrix in Equation (59) as $K_{zz}^{f}$, which is symmetric, i.e.,

(60) $$K_{zz}^{f}=\left[\begin{array}{cccccc} k_{11}^{f} & k_{12}^{f} & k_{13}^{f} & -k_{11}^{f} & -k_{12}^{f} & k_{13}^{f}\\ k_{12}^{f} & k_{22}^{f} & k_{23}^{f} & -k_{12}^{f} & -k_{22}^{f} & k_{23}^{f}\\ k_{13}^{f} & k_{23}^{f} & k_{33}^{f} & -k_{13}^{f} & -k_{23}^{f} & k_{36}^{f}\\ -k_{11}^{f} & -k_{12}^{f} & -k_{13}^{f} & k_{11}^{f} & k_{12}^{f} & -k_{13}^{f}\\ -k_{12}^{f} & -k_{22}^{f} & -k_{23}^{f} & k_{12}^{f} & k_{22}^{f} & -k_{23}^{f}\\ k_{13}^{f} & k_{23}^{f} & k_{36}^{f} & -k_{13}^{f} & -k_{23}^{f} & k_{33}^{f} \end{array}\right],$$

The nonzero elements in the stiffness matrix $K_{zz}^{f}$ are

(61) $$\begin{array}{c} k_{11}^{f}=768R_{1}R_{2}\cos^{3}\theta (12R_{2}\sin^{2}\theta +R_{1}L^{2})/rL,\\ k_{22}^{f}=\left[9216R_{1}{\left(R_{2}\right)}^{2}\cos^{5}\theta +192{\left(R_{1}\right)}^{2}R_{2}L^{2}\sin^{2}\theta \cos\theta \right]/rL,\\ k_{12}^{f}=-288{\left(R_{1}\right)}^{2}R_{2}L\sin\theta \cos^{2}\theta /r,\\ k_{33}^{f}=R_{2}\cos\theta \left[\begin{array}{c} 192R_{1}R_{2}L^{2}\cos2\theta -9216{\left(R_{2}\right)}^{2}\sin^{2}\theta \cos^{4}\theta \\ -3264R_{1}R_{2}L^{2}\cos^{4}\theta -55{\left(R_{1}\right)}^{2}L^{4}\sin^{2}\theta \end{array}\right]/rL,\\ k_{13}^{f}=k_{12}^{f}L/2,\\ \begin{array}{c} k_{23}^{f}=k_{22}^{f}L/2,\\ k_{36}^{f}=-k_{33}^{f}+k_{22}^{f}L^{2}/2, \end{array} \end{array}$$

where $R_{1}$ and $R_{2}$ are coefficients related to the materials and the cross-section of the piezoelectric laminated zigzag beam: $R_{1}=Y_{p}A_{p}+Y_{s}A_{s}$ and $R_{2}=Y_{p}I_{p}+Y_{s}I_{s}$. Neglecting the internal structure and dimensions at the nodes, L is the length of the piezoelectric laminated zigzag beam, which can be express as

(62) L=4lcosθ,

and the value of r is

(63) $$r=\begin{array}{c} \sin^{2}\theta \left[7{\left(R_{1}\right)}^{2}L^{4}+9216{\left(R_{2}\right)}^{2}\cos^{4}\theta \right]-\\ 192R_{1}R_{2}L^{2}\cos\left(2\theta \right)+960R_{1}R_{2}L^{2}\cos^{4}\theta \end{array}.$$

## 3. Macroscopic Mechanical Properties of the Lattices

Since the lattice is a periodic structure, the deformation of a single unit cell can be used to represent the overall deformation of the lattice. Assume that the deformations of the unit cell are all small deformations within the range of linear elasticity. Then, the deformation of the lattice is composed of the superposition of the deformation caused by the applied force and the deformation caused by the voltage.

Before deriving the macroscopic modulus of the lattice, we first establish a piezoelectric laminated zigzag beam square lattice in the finite element software (COMSOL Multiphysics 6.1) and apply a voltage or a uniform horizontal tensile force to preliminarily observe the deformation trend of the lattice, providing a basic idea for the force and deformation analysis of the unit cell. The deformation of the lattice is found to be periodic. When the lattice is subjected to a tensile force, it not only undergoes tensile deformation but also downward shear deformation, indicating that this type of lattice exhibits a dilatancy effect [18]. However, subsequent finite element results show that the influence of dilatancy on the calculation of the Young’s modulus of the lattice is negligible. Therefore, in the following derivation, the effect of dilatancy on the Young’s modulus is ignored, and the force–displacement relationship of a single zigzag beam under tension at both ends is used for calculation. Hence, this section attempts to approximate the deformation of the lattice using the deformation of the piezoelectric laminated zigzag beam that constitutes it.

### 3.1. Young’s Modulus

**Figure 7:**
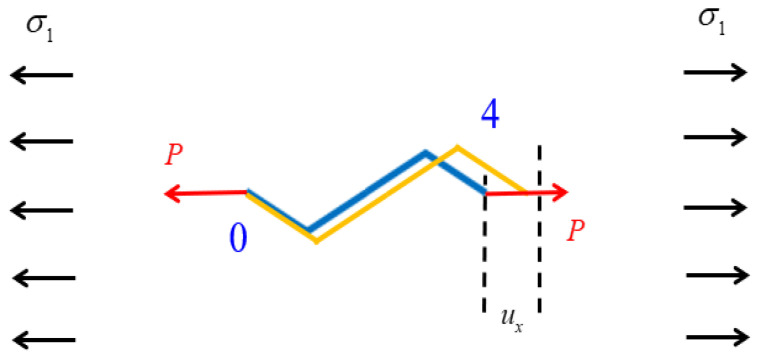
Tensile deformation of piezoelectric laminated zigzag beams.

In Figure 2b, nodes 0 and 4 are connected to the centers of two unit cells. Therefore, the deformation of this section of the piezoelectric laminated zigzag beam can be used to represent the deformation of the computational unit cell. In Figure 7, when uniform stress along the *x* direction is applied to the lattice, the force acting on a single unit cell is

(64) $$P=\sigma_{1}Lb,$$

The deformation of the piezoelectric zigzag beam is generated by the superposition of the action of force and the action of the voltage.

The displacements $d_{0}^{f}$ and $d_{4}^{f}$ caused by $\sigma_{1}$ can be obtained via Equation (59):

(65) $$\left[\begin{array}{c} d_{0}^{f}\\ d_{4}^{f} \end{array}\right]={\left[\begin{array}{cc} K_{1}^{f} & K_{2}^{f}\\ K_{3}^{f} & K_{4}^{f} \end{array}\right]}^{-1}\left[\begin{array}{c} F_{0}\\ F_{4} \end{array}\right],$$

where $d_{0}^{f}={\left[\begin{array}{ccc} 0 & 0 & w_{0}^{f} \end{array}\right]}^{t}$ and $d_{4}^{f}={\left[\begin{array}{ccc} u_{4}^{f} & v_{4}^{f} & w_{4}^{f} \end{array}\right]}^{t}$. $u_{4}^{f}$ and $v_{4}^{f}$ represent the displacement caused by the force in the *x* and *z* direction at node 4, respectively; $w_{0}^{f}$ and $w_{4}^{f}$ represent the angles of rotation caused by the force at node 0 and node 4, respectively, and they satisfy the condition $w_{0}^{f}=w_{4}^{f}$. $F_{0}={\left[\begin{array}{ccc} P & 0 & 0 \end{array}\right]}^{t}$, and it is the external load at node 0; $F_{4}={\left[\begin{array}{ccc} P & 0 & 0 \end{array}\right]}^{t}$, and it is the external load at node 4.

The displacements $d_{0}^{U}$ and $d_{4}^{U}$ caused by *U* can be obtained from Equation (56):

(66) $$\left[\begin{array}{c} d_{0}^{U}\\ d_{4}^{U} \end{array}\right]={\left[\begin{array}{cc} K_{1} & K_{2}\\ K_{3} & K_{4} \end{array}\right]}^{-1}\left[\begin{array}{c} C_{0}\\ C_{4} \end{array}\right],$$

where $d_{0}^{U}={\left[\begin{array}{ccc} 0 & 0 & w_{0}^{U} \end{array}\right]}^{t}$ and $d_{4}^{U}={\left[\begin{array}{ccc} u_{4}^{U} & 0 & w_{4}^{U} \end{array}\right]}^{t}$. $u_{4}^{U}$ is the displacement caused by the voltage in the *x* direction at node 4; $w_{0}^{U}$ and $w_{4}^{U}$ represent the angles of rotation caused by the voltage at node 0 and node 4, respectively, and they also satisfy the condition $w_{0}^{U}=w_{4}^{U}$; $C_{0}$ and $C_{4}$ are given in Equation (55).

The displacements of the two nodes under the combined action of stress and voltage are

(67) $$d_{0}=d_{0}^{f}+d_{0}^{U},$$

(68) $$d_{4}=d_{4}^{f}+d_{4}^{U},$$

The elongation of the unit cell in the *x*-direction can be expressed as

(69) $$u_{x}=u_{4}^{f}+u_{4}^{U},$$

The angle of rotation at nodes 0 and 4 can be expressed as

(70) $$w=w_{4}^{f}+w_{4}^{U}.$$

The strain of the unit cell in the *x*-direction can be expressed as

(71) $$\varepsilon_{x}=\frac{u_{x}}{L},$$

Finally, the Young’s modulus of the square lattice composed of piezoelectric laminated zigzag beams in the *x* direction can be obtained as

(72) $$E_{x}=\frac{\sigma_{x}}{\varepsilon_{x}}.$$

Since the structure of the lattice in the *x* direction is the same as that in the *z*-direction, when uniform stress in the *z* direction is applied to the lattice, the deformation generated by the lattice is the same as that when stress in the *x*-direction is applied. Therefore, the Young’s modulus of the square lattice composed of the piezoelectric laminated zigzag beams in the *z* direction is

(73) $$E_{z}=E_{x}.$$

To ensure that the deformation of the piezoelectric laminated zigzag beam can be used to substitute the deformation of the lattice and thereby calculate the macroscopic modulus of the lattice, we compare the relative displacement between two adjacent nodes in the lattice from finite element results with the method proposed in this section. When the voltage applied to the piezoelectric laminated zigzag beam is 40 V and the uniform tensile stress is 400 Pa, Table 1 shows the relationship between the displacements obtained by the two methods and the angle between each component of the piezoelectric laminated zigzag beam and the horizontal direction. Within an inclination angle of $50^{\circ }$, the relative difference between the two is within 2%, so the calculation method proposed in this section can be used to approximately calculate the macroscopic modulus of the lattice.

### 3.2. Poisson’s Ratio

In the study of the macroscopic mechanical properties of square lattice composed of zigzag beams [18], which is composed of a single material, the Poisson’s ratio of the lattices composed of the zigzag beam in the *x* direction is zero. This means that the force in the *x* direction will not have an impact on the deformation in the *z* direction. Therefore, when the voltage is zero, the square lattice composed of piezoelectric laminated zigzag beams has the same macroscopic mechanical properties as that of the square lattice composed of zigzag beams. That is, when only $\sigma_{x}$ is applied, there will be no strain in the *z* direction due to the force, while the voltage will cause deformations in both the *x* and *z* directions of the lattice material. When the unit cell is under the combined action of the stress $\sigma_{x}$ and the voltage *U*, the deformation of the unit cell in the *x* direction is generated by the superposition of the stress and the voltage. Therefore, the strain of the unit cell in the *x* direction is given by Equation (71). The deformation of the unit cell in the *z* direction is only caused by the voltage, and the structures of the lattice in the two directions are completely the same. To reduce the amount of calculation, $u_{4}^{U}$ can be used to represent the deformation of the unit cell in the *y*-direction. Therefore, the strain $\varepsilon_{z}$ of the unit cell in the *z* direction can be expressed as

(74) $$\varepsilon_{z}=\frac{u_{4}^{U}}{L}.$$

According to the definition of Poisson’s ratio, the Poisson’s ratio of the square lattice composed of piezoelectric laminated zigzag beams in the *x*-direction can be expressed as

(75) $$v_{xz}=-\frac{\varepsilon_{z}}{\varepsilon_{x}}=-\frac{u_{4}^{U}}{u_{4}^{U}+u_{4}^{f}}.$$

It can be known from the above equation that when the voltage is zero, the displacement caused by the voltage is zero, and at this time, $v_{xz}=0$, which is consistent with the square lattice [18] of zigzag beams composed of a single homogeneous material. This proves the accuracy of the calculation method adopted in this paper. Since the structures in the *x*-direction and the *z* direction are equal, the Poisson’s ratio of the square lattice composed of piezoelectric zigzag beams in the *z* direction is

(76) $$\nu_{zx}=v_{xz}.$$

### 3.3. Shear Modulus

**Figure 8:**
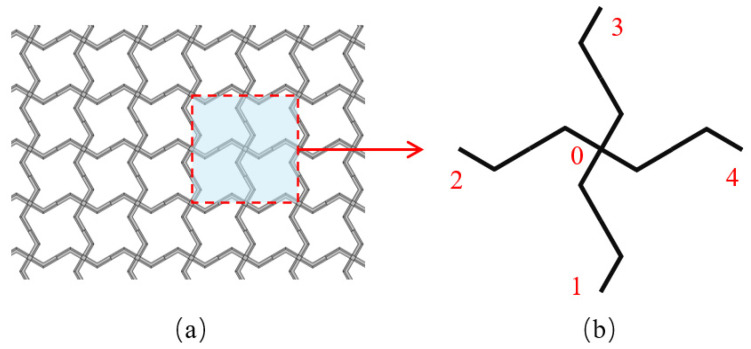
The square lattice composed of the piezoelectric laminated zigzag beams: (**a**) structure; (**b**) shear modulus analysis unit.

**Figure 9:**
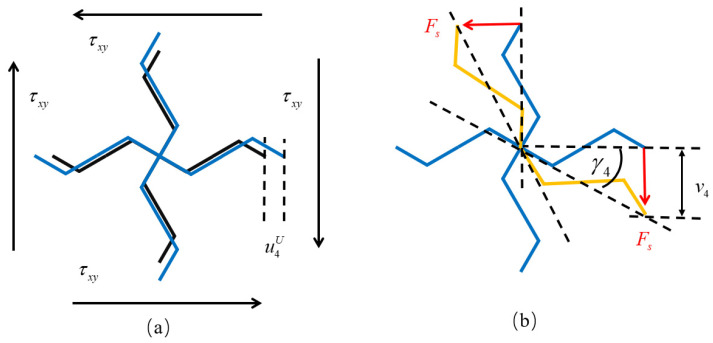
Shear deformation: (**a**) deformation caused by voltage; (**b**) deformation caused by $\tau_{12}$.

Apply both the shear stress $\tau_{xz}$ and the voltage U to the lattice simultaneously. Take the structure selected in Figure 8a as a basic unit. Assume that the displacement at the central node 0 is zero. The unit cell expands or contracts under the action of the voltage, and the deformed unit cell caused by the voltage is shown in Figure 9a. Then, the displacement of node 4 relative to node 0 caused by the voltage can be obtained from Equation (56), and it can be express as

(77) $$d_{4}^{U}={\left[\begin{array}{ccc} u_{4}^{U} & 0 & w_{4}^{U} \end{array}\right]}^{t}.$$

.

The shear force at node 4 is

(78) $$F_{s}=2\tau_{xz}Lb,$$

The displacement of node 4 relative to node 0 caused by the shear force can be obtained from Equation (59), and it can be express as

(79) $$\left[\begin{array}{c} u_{4}^{f}\\ v_{4}^{f}\\ w_{4}^{f} \end{array}\right]={\left(K_{4}^{f}\right)}^{-1}\left[\begin{array}{c} 0\\ F_{s}\\ 0 \end{array}\right].$$

Figure 9b shows the deformation of the unit cell caused by the shear force. The black lines represent the undeformed structure, the blue lines represent the structure after deformation due to voltage application, and the yellow lines represent the structure after deformation caused by force. Then, under the combined action of the voltage and the shear force, the deflection angle of beam segment 04 can be expressed as

(80) $$\gamma_{4}=\frac{v_{4}^{f}}{L+u_{4}^{f}+u_{4}^{U}},$$

Due to the central symmetry of the lattice structure, the deformation of beam segment 03 under the action of the shear force is opposite to that of beam segment 04, so the deflection angle of beam segment 03 can be expressed as

(81) $$\gamma_{3}=\frac{v_{4}^{f}}{L-u_{4}^{f}+u_{4}^{U}}.$$

According to the definition of the shear modulus, the shear modulus of the square lattice composed of the piezoelectric laminated zigzag beams is

(82) $$G=\frac{\tau_{xz}}{\gamma_{3}+\gamma_{4}}.$$

## 4. Comparative Verification of the Finite Element Method

We use the software MATLAB (R2022a) to obtain the above macroscopic moduli and conduct simulation using the finite element software COMSOL 6.1 for comparisons. The relevant parameters of the model are given in Table 2.

**Figure 10:**
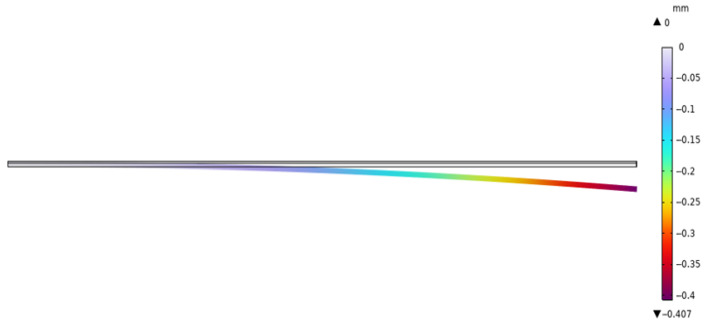
The deflection of the piezoelectric laminated cantilever beam.

**Figure 11:**
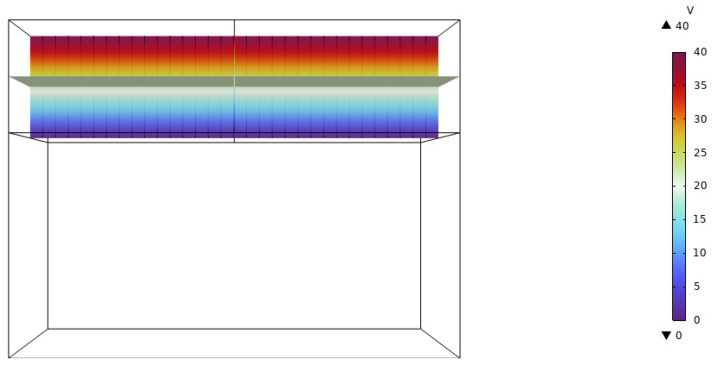
The voltage distribution of the piezoelectric laminated cantilever beam.

**Figure 12:**
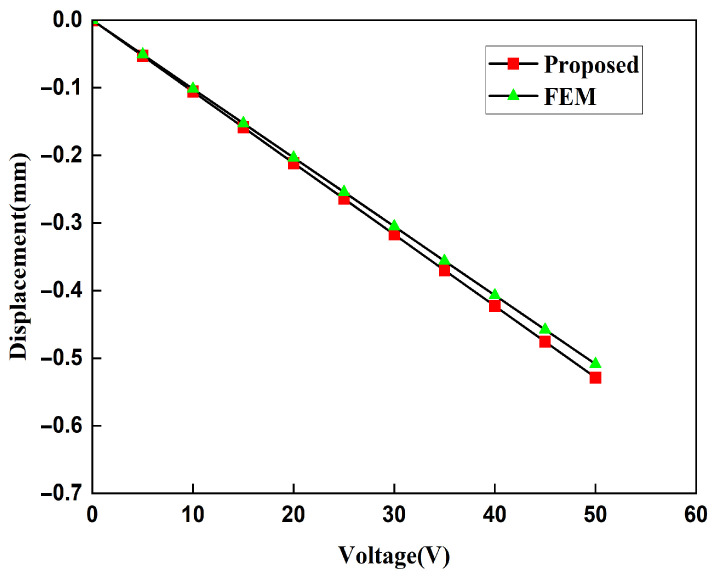
Comparison of displacements of the single-layer piezoelectric cantilever beam.

First, we established a model of the piezoelectric laminated straight beam, and we took the cantilever beam as an example to verify the accuracy of $\left[K_{0}\right]$. Its deformation diagram is shown in Figure 10, and the voltage is shown in Figure 11. The displacements of its free end obtained by the two methods are shown in Figure 12. Under the conditions of the same size and material, both the finite element results and the theoretical results are consistent with existing studies [34]. When the voltage is 50 V, the free end’s displacement values obtained by our two methods (0.50872 mm and 0.52867 mm) differ from those in existing studies (0.52867 mm) by 3.77%, and the theoretical solution is consistent with existing studies. Then, we established a model of the piezoelectric laminated zigzag beam to verify the accuracy of $K_{zz}^{U}$.

In our model, we established a solid element model and realized the coupling of force and electricity by adding multi-physics fields and piezoelectric effects. The model is set up in layers and assigned different materials. The mesh is divided into hexahedral solid elements by swept (equivalent to solid 186 in ANSYS 2023 R1). The boundary conditions applied in the COMSOL are as follows: The displacements in both the *x* and *z* directions are constrained on the left boundary (node 0) of the model, but rotation is allowed; on the right boundary (node 4), the displacement in the *z* direction is constrained, enabling only displacement and rotation in the *x* direction.

**Figure 13:**
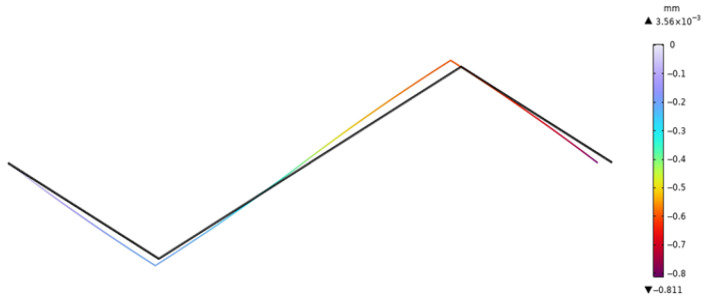
The displacement in the *x* direction of the piezoelectric laminated zigzag beam.

**Figure 14:**
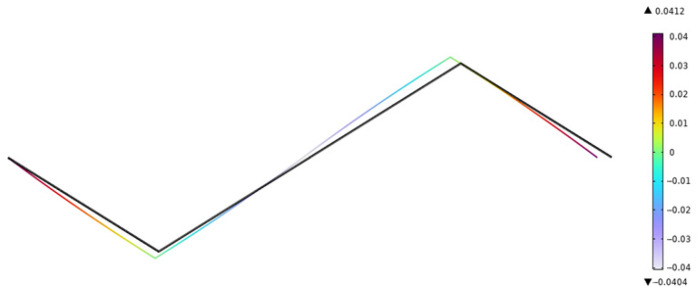
The rotation angle of the piezoelectric laminated zigzag beam.

By adjusting the voltage variation, the displacement value and rotation angle at the node on the right end are extracted. The deformation diagram of the *x* component of the piezoelectric laminated zigzag beam when the voltage is 40 V is shown in Figure 13. It can be seen from Figure 13 that the deformation of the piezoelectric laminated zigzag beam is symmetrically distributed about the center. The rotation angle of the piezoelectric laminated zigzag beam is shown in Figure 14. It can be seen from Figure 14 that the rotation angles at the nodes at both ends are equal, and they are opposite in value to the value at the middle node. When the voltage changes, the displacements and rotation angles of the node at the right end of the piezoelectric laminated zigzag beam obtained by the two methods are shown in Table 3. This verifies the correctness of the theoretical derivation of the relationship between the voltage load and the displacement of the piezoelectric laminated zigzag beam.

**Figure 15:**
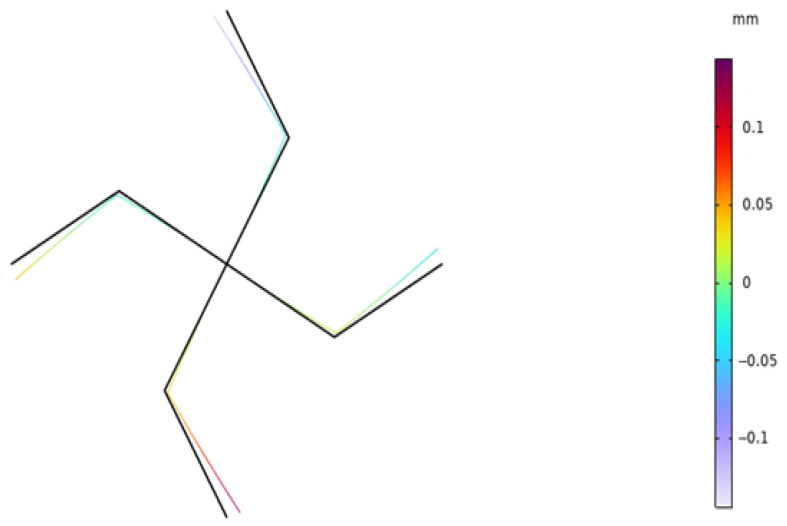
Displacement diagram of the unit cell of the square lattice composed of piezoelectric laminated zigzag beams in the *x* direction (under voltage).

**Figure 16:**
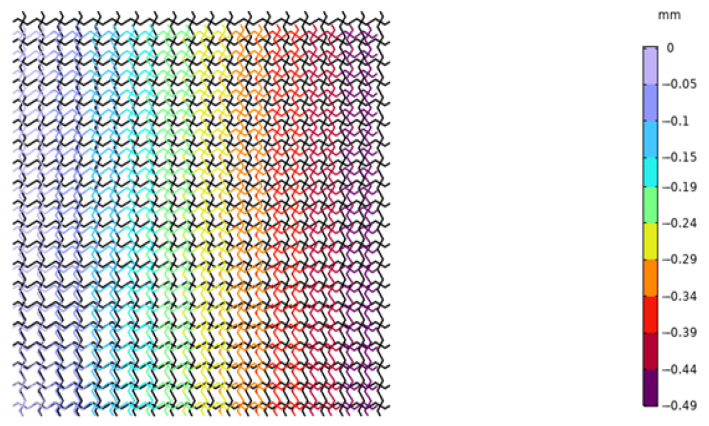
Displacement diagram of the square lattice composed of piezoelectric laminated zigzag beams in the *x* direction (under force).

**Figure 17:**
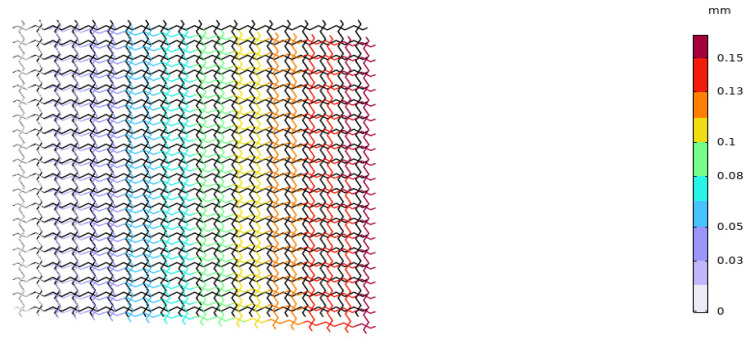
Displacement diagram of the square lattice composed of piezoelectric laminated zigzag beams in the *x*-direction.

We applied the same boundary conditions to the unit cells of the lattice composed of the piezoelectric laminated zigzag beams in COMSOL. The displacement of the unit cell in the *x* direction when the voltage is 4 V is shown in Figure 15. It can be seen in Figure 15 that the deformation of the zigzag beam in the *z* direction is the same as that in the *x* direction. Subsequently, when *l* = 1, we established a 20 × 20 square lattice. The displacement diagram of this lattice in the *x* direction when the voltage is 500 V is shown in Figure 16. It can be observed in Figure 16 that the lattice exhibits a contraction deformation under the action of positive voltage. Figure 17 shows the deformation diagram of the lattice under tensile force. It can be seen that the lattice not only undergoes tensile deformation in the *x* direction but also produces downward shear deformation, which corresponds to the tension–shear coupling characteristic [18] of chiral lattices.

**Figure 18:**
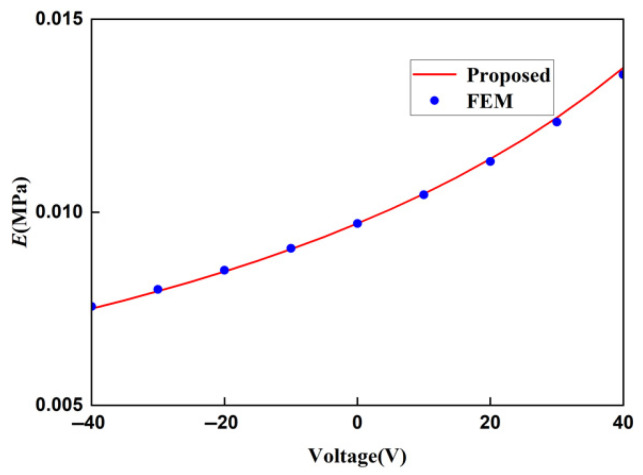
Comparison of theoretical and FEM results for the Young’s modulus of square lattices composed of piezoelectric laminated zigzag beams.

**Figure 19:**
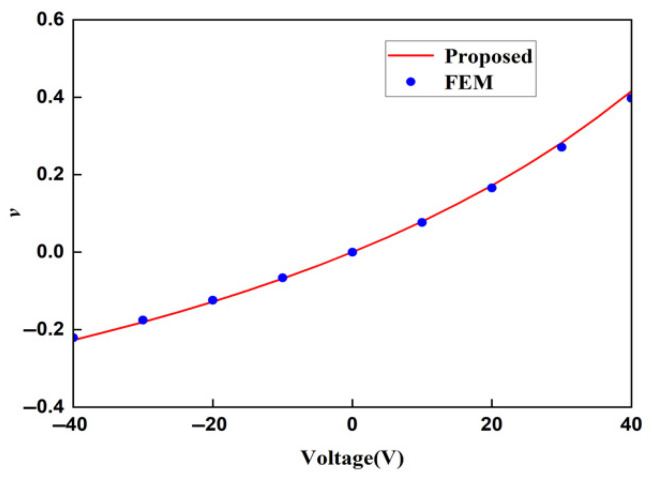
Comparison of theoretical and FEM results for the Poisson’s ratios of square lattices composed of piezoelectric laminated zigzag beams.

**Figure 20:**
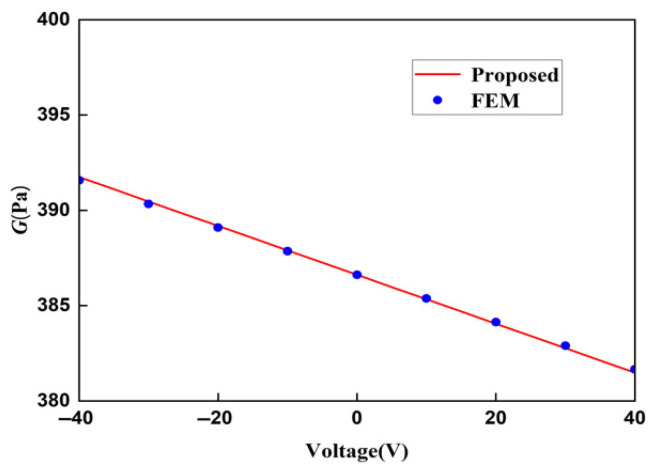
Comparison of theoretical and FEM results for the shear modulus of square lattices composed of piezoelectric laminated zigzag beams.

The displacement obtained by the finite element method can be used to calculate the strain of the lattice, and then, the Young’s modulus of the lattice can be calculated. In order to ensure the accuracy of the finite element results, when the voltage applied to the lattice is 20 V and the stress is 400 MPa, the Young’s moduli of the lattice under different mesh precisions are shown in Table 3. It can be concluded from Table 4 that the Young’s moduli obtained from the finer mesh and the extra fine mesh tend to converge. This is to say that the results obtained from the extra-fine mesh can be used to represent the solution of the finite element method. Using a mesh of finer we change the applied voltage and compare the macroscopic modulus obtained from the finite element method with that obtained from the theoretical formula. The comparison diagrams of the Young’s modulus, Poisson’s ratio, and shear modulus of the lattice obtained by the two methods are shown in Figure 18, Figure 19, and Figure 20, respectively. It can be seen from the above three figures that the results of the two are similar, which verifies the correctness of the derivation and the calculation of the macroscopic modulus of the square lattice composed of piezoelectric laminated zigzag beams.

In the above three figures, when the voltage is zero, the mechanical properties of the lattice obtained by the two methods are equal because there is no piezoelectric effect at this time. When the absolute value of the applied voltage is larger, the error between the finite element result and the theoretical result is greater. This also follows the same pattern as the error in the displacement of the piezoelectric zigzag beam in Table 2. Therefore, it can be inferred that the error originates from the deformation caused by the piezoelectric effect.

## 5. Numerical Results and Analyses

Through the previous theoretical derivations, we have obtained the macroscopic mechanical properties of the square lattice composed of piezoelectric laminated zigzag beams, and we have verified it in COMSOL. It is evident that its Young’s modulus, Poisson’s ratio, and shear modulus all change with variations in voltage. Next, we will conduct a detailed exploration of the mechanism by which voltage influences its mechanical properties, thereby providing guidance for us to regulate the mechanical properties of the lattice using voltage.

### 5.1. Young’s Modulus

Through the above research, we have found that the Young’s modulus of the square lattice composed of the piezoelectric laminated zigzag beams varies with the voltage. Consequently, the stiffness of the lattice can be adjusted by changing the voltage. Three stresses of different magnitudes are applied transversely to the lattice, and the voltage is changed simultaneously so as to obtain the influence law of the voltage and external forces on Young’s modulus.

**Figure 21:**
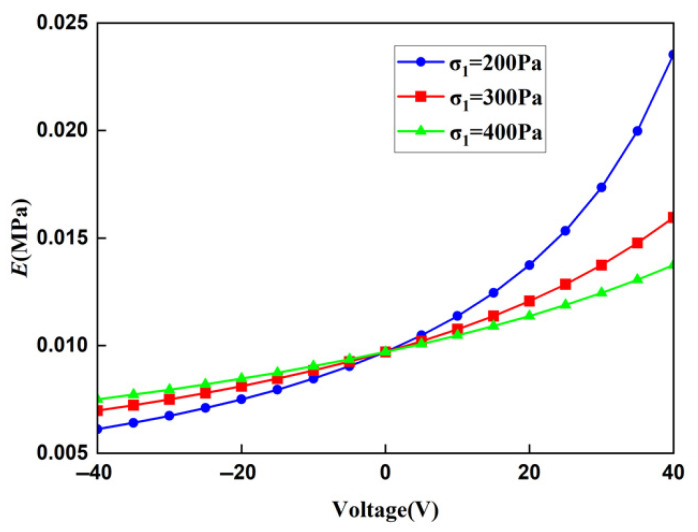
The relationship between the macroscopic Young’s modulus of the square lattice composed of piezoelectric laminated zigzag beams and the voltage.

The relationship between the macroscopic Young’s modulus of the lattice and the voltage under different stress fields is shown in Figure 21. When the voltage is zero, the three curves intersect at the same point. At this time, it is equivalent to a common composite material beam lattice, and its Young’s modulus is a fixed value, which is also consistent with research [18]. When the stress applied to the lattice remains unchanged, the macroscopic Young’s modulus of the lattice increases as the voltage increases. When the applied voltage is constant, the macroscopic Young’s modulus of the lattice is related to the magnitude of the stress. Taking zero voltage as the demarcation point, when the voltage is positive, stress is lower, and Young’s modulus is higher; when the voltage is negative, there is greater stress, and Young’s modulus is higher. This is caused by the contraction or expansion of the lattice under the action of the voltage. A positive voltage will cause the lattice to contract, reducing the total deformation of the lattice. When stress is greater, the proportion of the deformation caused by the voltage in the total deformation gradually decreases, and the ability to regulate the lattice also decreases accordingly. The same principle applies when the voltage is negative. Therefore, the greater the stress, the smaller the variation range of the curve.

By using this rule, the magnitude of the voltage value can be adjusted according to the external load applied to the lattice so that the lattice can be in an appropriate stiffness state. By taking advantage of this rule, the Young’s modulus of the lattice can be effectively controlled. This lattice can be used to sense environments and develop biomedical devices. The mechanical energy in the environment is converted into electrical energy through the piezoelectric effect of piezoelectric lattice materials with respect to power sensors, enabling long-term monitoring without batteries. The flexible piezoelectric lattice film is attached to the surface of the heart, generating electricity through heartbeat vibrations while monitoring heart rate, myocardial contractility, and other parameters. The deformation of the piezoelectric lattice structure is triggered by external electrical stimulation or in vivo mechanical signals (such as changes in blood pressure), thereby controlling the opening and closing of drug release channels.

The periodic structure of piezoelectric lattice metamaterials endows them with unique capabilities to modulate the propagation of elastic waves (stress waves and acoustic waves). Moreover, the adjustable stiffness properties can further amplify the signal characteristics of defect regions, enhancing the detection sensitivity for defect identification and structural integrity monitoring. This not only provides a new approach for the dynamic non-destructive monitoring of composite structures under complex working conditions but also further highlights the cross-scenario potential of this model from mechanical regulation to diagnostic applications.

### 5.2. Poisson’s Ratio

**Figure 22:**
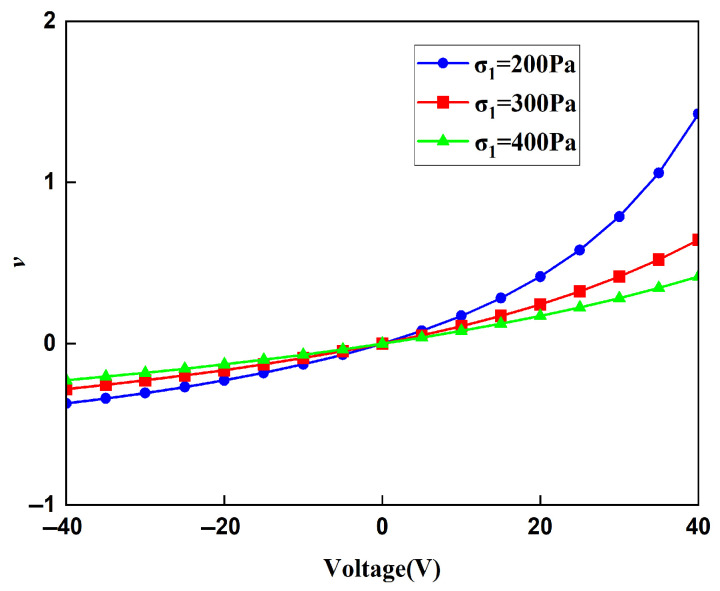
The relationship between the macroscopic Poisson ratios of the square lattice composed of piezoelectric laminated zigzag beams and the voltage.

The relationship between the Poisson ratio of the lattice and the voltage under different stress fields is shown in Figure 22. The Poisson ratio of the lattice in the x direction has similar properties to Young’s modulus, and Poisson’s ratio increases as the voltage increases. When the voltage is zero, regardless of the magnitude of the applied stress, the Poisson ratio is always zero, which is the same as the conclusion of the Poisson ratio of the lattice composed of zigzag beams without piezoelectric materials; that is, the stretching in the *x* direction will not cause expansion and contraction changes in the *z* direction. When the voltage gradually increases due to the deformation of the piezoelectric zigzag beams in the *z* direction under the action of the voltage, the lattice thus has strain in the *z* direction. Therefore, Poisson’s ratio is no longer zero, breaking the rule that the Poisson ratio of the lattice composed of zigzag beams with traditional materials is zero.

The influence mechanism of voltage on the Poisson ratio of the lattice is the same as that on Young’s modulus. By adjusting the voltage, the elongation or contraction of the horizontal and vertical zigzag beams in the lattice can be regulated, thereby adjusting the magnitude of the Poisson ratio of the lattice. Similarly to the Young’s modulus, for the lattice under the same voltage, the greater the stress, the closer the value of the Poisson ratio to zero, and the less obvious the regulation of Poisson’s ratio by the voltage. When the external load remains constant, the greater the voltage, the more obvious its regulation effect on Poisson’s ratio.

Deformable materials have been widely used in the medical field, such as shape-memory alloy vascular stents [49] and stents with adjustable sizes [50]. The lattice structure with precisely controllable deformation proposed in this paper provides the possibility of enhancing biomedical adaptability and control accuracy. Its precise and adjustable deformation capability makes it possible to design sensors with smaller volume and higher precision. However, the implications of underground stress fields, energy localization in beams, and surface defects have not yet been explored. Research on lattice defects [51] provides experience and methods for exploring the impact of defects on the performance of lattices.

### 5.3. Shear Modulus

**Figure 23:**
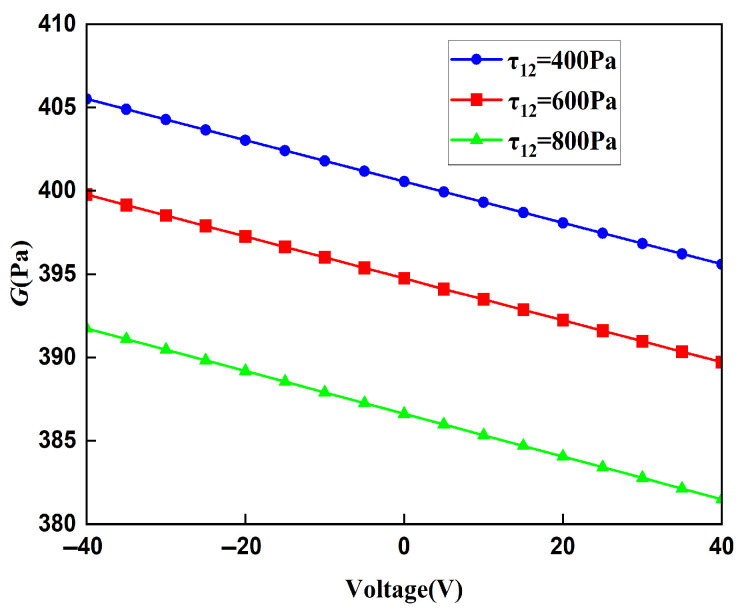
The relationship between the macroscopic shear modulus of the square lattice composed of piezoelectric zigzag beams and the voltage.

The relationship between the shear modulus of the lattice and the voltage under different shear stress fields is shown in Figure 23. When shear stress remains constant, the shear modulus shows a decreasing trend as the voltage increases. And when the voltage is constant, the magnitude of the shear modulus is related to the magnitude of the shear stress. The greater the shear stress, the lower the shear modulus. Different from Young’s modulus and Poisson’s ratio, when the voltage is zero, the shear modulus is not a fixed value, and it is still related to the magnitude of the shear stress applied. The reason for this phenomenon is that the lattice composed of zigzag beams has a tensile–shear coupling effect. Its shear deformation will cause the zigzag beam to undergo expansion and contraction deformation. Due to the reciprocal theorem of shear stress, the directions of the shear stresses applied to the piezoelectric zigzag beams in the horizontal direction and the vertical direction are opposite, resulting in two opposite contraction states, which in turn affect the size of the unit cell after deformation, making the shear modulus change with the shear stress.

## 6. Conclusions

We proposed a design scheme and theoretical model for a square lattice composed of piezoelectric laminated zigzag beams positioned between every pair of adjacent nodes. Using the derived stiffness matrix of these beams, we analyze their deformation. The effective moduli are derived by analyzing the unit cell under a stress field. Compared with finite element simulations based on linear solid models, our method accurately predicts the macroscopic mechanical properties of this piezoelectric lattice structure. Parametric analysis is adopted to study the effects of voltage and external stress fields on the lattice’s effective moduli.

The proposed lattice exhibits a wide adjustable modulus range. By controlling voltage, it achieves both high strength and a negative Poisson ratio. These properties not only demonstrate voltage-dependent material behavior but also inspire new designs for smart materials and tunable structures. Although this study focuses on square lattices, the model extends to other actuation modes, such as magnetic fields, hydraulics, and pneumatics, as well as diverse lattice types, including honeycomb and triangular configurations. Additionally, beam geometry variations (e.g., curved shapes) can further enhance mechanical performance and broaden engineering applications.

This piezoelectric metamaterial shows significant potential across multiple fields. In aerospace, it enables adjustable stiffness components for different flight phases and lightweight high-strength structures. For robotics, adaptive lattice structures improve system functionality and adaptability. In smart buildings, dynamic structural adjustments enhance stability and system intelligence. Notably, when voltage exceeds a threshold, large beam deformations must be considered due to their critical impact on structural performance. This effect will be a key focus of future research.

This adjustable lattice has great application prospects. It can be used in many fields such as architectural and bridge structures, biomedical devices, structural health monitoring, and electronic sensors.

The model proposed in this paper still has many limitations. For example, the proposed theoretical models are all within the range of linear elastic deformations and will no longer be applicable when the structure undergoes large deformations. The performance of the structure under different temperature environments and the fatigue or degradation of the piezoelectric response during cycling are not considered. In addition, in subsequent studies, experiments can also be designed to further analyze the mechanical properties of the structure. These issues warrant deeper exploration.

## Figures and Tables

**Table 1:** The relationship between the displacement in the *x*-direction of the piezoelectric laminated zigzag beam and the angle of the zigzag beam.

Angle θ(°)	FEM Displacement (mm)	Proposed Displacement (mm)
0	0.008615	0.008532
10	0.1074	0.1062
20	0.897	0.8789
30	2.044	2.016
40	3.128	3.093
50	3.726	3.683

**Table 2:** The dimensional parameters and material properties of the square lattice composed of piezoelectric laminated zigzag beams.

Parameter	Value
Length of piezoelectric layer *l* (mm)	20
Width of piezoelectric layer *b* (mm)	0.2
Thickness of piezoelectric layer *h_p_* (mm)	0.05
Young’s modulus of piezoelectric layer *E_p_* (N/m^2^)	$1.27\times 10^{11}$
Piezoelectric constant *d*_31_	$-2.74\times \;10^{-10}$
Relative permittivity ζ	1433.6
Length of substrate layer *l* (mm)	20
Width of piezoelectric layer *b* (mm)	0.2
Thickness of piezoelectric layer *h_s_* (mm)	0.1
Young’s modulus of piezoelectric layer *E_s_* (N/m^2^)	$7\times 10^{10}$
Angle of the zigzag beam θ(rad)	π/6

**Table 3:** Comparison of the displacements of the node at the right end of the piezoelectric laminated zigzag beam.

Voltage (V)	Displacement (mm)	Displacement (mm)	Rotation Angle (Rad)	Rotation Angle (Rad)
	FEM	Proposed	FEM	Proposed
−40	0.8113	0.8386	0.0403	0.0423
−30	0.6085	0.629	0.0308	0.0317
−20	0.4057	0.4193	0.0205	0.0211
−10	0.2029	0.2097	0.0103	0.0106
0	0	0	0	0
10	−0.2029	−0.2097	−0.0103	−0.0106
20	−0.4057	−0.4193	−0.0205	−0.0211
30	−0.6085	−0.6290	−0.0308	−0.0317
40	−0.8113	−0.8386	−0.0403	−0.0423

**Table 4:** The accuracy of the finite element mesh and the Young’s modulus of the square lattice.

Mesh Precisions	Young’s Modulus (MPa)
Coarse	$1.1298\times \;10^{-2}$
Normal	$1.1305\times 10^{-2}$
Fine	$1.1310\times 10^{-2}$
Finer	$1.1314\times 10^{-2}$
Extra fine	$1.1314\times \;10^{-2}$

## Data Availability

The original contributions presented in this study are included in the article. Further inquiries can be directed to the corresponding author.
